# Opioid Deaths: Trends, Biomarkers, and Potential Drug Interactions Revealed by Decision Tree Analyses

**DOI:** 10.3389/fnins.2018.00728

**Published:** 2018-10-23

**Authors:** Manal H. Saad, Candace L. Savonen, Matthew Rumschlag, Sokol V. Todi, Carl J. Schmidt, Michael J. Bannon

**Affiliations:** ^1^Department of Pharmacology, Wayne State University School of Medicine, Detroit, MI, United States; ^2^Department of Pathology, University of Michigan Medical School, Ann Arbor, MI, United States; ^3^Wayne County Medical Examiner’s Office, Detroit, MI, United States

**Keywords:** CHAID, fentanyl, cocaine, heroin, morphine, methadone, acetaminophen, citalopram

## Abstract

Opioid abuse is now the primary cause of accidental deaths in the United States. Studies over several decades established the cyclical nature of abused drugs of choice, with a current resurgence of heroin abuse and, more recently, fentanyl’s emergence as a major precipitant of drug-related deaths. To better understand abuse trends and to explore the potential lethality of specific drug–drug interactions, we conducted statistical analyses of forensic toxicological data from the Wayne County Medical Examiner’s Office from 2012–2016. We observed clear changes in opioid abuse over this period, including the rapid emergence of fentanyl and its analogs as highly significant causes of lethality starting in 2014. We then used Chi-square Automatic Interaction Detector (CHAID)-based decision tree analyses to obtain insights regarding specific drugs, drug combinations, and biomarkers in blood most predictive of cause of death or circumstances surrounding death. The presence of the non-opioid drug acetaminophen was highly predictive of drug-related deaths, likely reflecting the abuse of various combined acetaminophen-opioid formulations. The short-lived cocaine adulterant levamisole was highly predictive of a short post-cocaine survival time preceding sudden non-drug-related death. The combination of the opioid methadone and the antidepressant citalopram was uniformly linked to drug death, suggesting a potential drug–drug interaction at the level of a pathophysiological effect on the heart and/or drug metabolism. The presence of fentanyl plus the benzodiazepine midazolam was diagnostic for in-hospital deaths following serious medical illness and interventions that included these drugs. These data highlight the power of decision tree analyses not only in the determination of cause of death, but also as a key surveillance tool to inform drug abuse treatment and public health policies for combating the opioid crisis.

## Introduction

Drug-related deaths now exceed all other causes of accidental death; the majority of these deaths involves opioid abuse (Centers for disease control and prevention, 2018). The past 15 years have seen sustained, parallel increases in prescription opioid sales, prescription opioid overdose deaths, and opioid treatment admissions, followed by a resurgence in heroin abuse and deaths and, most recently, a steep rise in opioid deaths involving fentanyl and fentanyl analogs (Kolodny et al., 2015; Rudd et al., 2016). In addition, the co-abuse of opioid and non-opioid drugs is the rule rather than the exception (Algren et al., 2013). In short, the pattern of opioid abuse and overdose deaths in the current epidemic is complex and evolving, rendering detection and intervention all the more difficult.

In the mid-2000s, the detection of a multistate pattern of fentanyl fatalities led to concerted public health and law enforcement responses believed to have cut short that outbreak (Algren et al., 2013). Forensic toxicological analyses by medical examiners can thus provide a crucial sentinel surveillance function for the community. In the present study, we examined post-mortem toxicological data from 7731 cases from the Wayne County Medical Examiner’s Office (WCMEO) covering the period of 2012–2016. Over this timespan, striking changes were evident in both the nature of opioids detected and the pattern of drugs co-abused. We used Chi-square Automatic Interaction Detector (CHAID) analyses (Kass, 1980) to build unbiased decision trees that predict the relationship between the presence of a given drug and the likelihood of case classification as a drug-related or non-drug-related death. Our results support the use of post-mortem drug screenings not only as an aid in the medico-legal determination of individual cause of death, but also as a sentinel surveillance tool to inform treatment and public health policies.

## Materials and Methods

The WCMEO conducts medico-legal investigations to determine the cause and manner of death in fatalities resulting from violence, in persons without recent medical attendance, and under other circumstances as outlined in the laws of the State of Michigan. The WCMEO makes such determinations on the basis of medical, police and other records, scene investigations, and autopsies, including pathology and toxicology findings. Death investigations by the WCMEO are mandated by law and not subject to review by an Institutional Review Board.

Comprehensive toxicological analyses were performed for the WCMEO by NMS labs^1^ under contract. WCMEO 2012–2016 cases with quantifiable values for at least one drug or other chemical compound in blood (*N* = 7731) were included in the study; of these, 2873 were drug-related deaths and 4858 were non-drug deaths, as determined by the WCMEO.

Statistical analyses were conducted using IBM SPSS Statistics version 24. Quantitative toxicological results for each drug were transformed into binary variables of 0 (for a negative finding) or 1 (for a positive finding, signifying the presence of a drug and/or its metabolites). These values were organized into a matrix that included de-identified case classification as drug death or non-drug deaths, which was then used for statistical analysis with an initial focus on changes in the prevalence of different drugs of abuse over time and the extent of co-abuse in drug deaths.

To evaluate the predictive power of drug-positive toxicology in the classification of cases as drug deaths vs. non-drug deaths, IBM SPSS Decision Tree Analysis was performed using the Pearson CHAID method. CHAID allows for the explorations of relationships between multiple predictor binomial variables (e.g., the presence or absence of a drug) and a categorical response variable (e.g., cause of death); it does so by building decision trees using chi-squared statistics that model these relationships hierarchically and provide an organized and interpretable visual representation of interactions within the data. In the present study, this method of analysis was used to identify individual drugs and drug combinations that were most significant in classifying drug-related deaths. Bonferroni-adjusted *p*-values were used to determine statistical significance (minimally *p* < 0.05). In addition to this statistical cut-off, decision tree settings used were: parent node sizes of ≥20, child node sizes of ≥10, and tree depth of 6 branches. Statistical findings are derived from the large intact trees presented in Supplementary Figures S1, S2; Figures 2, 3 show trees truncated to 4 branches and split into 2 sections after the first branch for ease of visualization. The placement of a particular branch to the right vs. left of a parent node is automatically set by the CHAID program, but is of no particular significance.

## Results and Discussion

**FIGURE 1:**
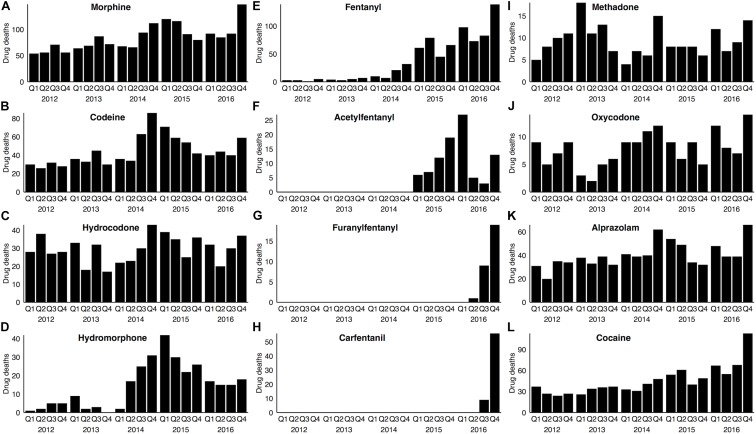
Opioid drugs associated with drug-related deaths in Wayne County from 2012–2016. In each panel, the number of drug deaths associated with a specific opioid drug **(A–J)**, or with alprazolam **(K)** or cocaine **(L)**, is represented on a quarterly basis. Note that the Y axis in each panel has been modified for maximum legibility. Drug deaths were determined based on medico-legal investigation and forensic toxicological data (see section “Materials and Methods”).

To first ascertain the drugs most commonly associated with drug-related deaths for 2012–2016, and to understand how stable the patterns of drug abuse were over time, we examined toxicological data from all drug-related deaths in Wayne County, Michigan during this time period, as described in the Materials and Methods. Figure 1 summarizes (by quarter) the number of cases determined positive for various opioids, as well as cocaine and alprazolam. As shown in Figure 1A, morphine was the opioid most commonly detected in drug death cases during this period. Importantly, the number of morphine-positive cases rose steadily, culminating in a near-doubling of such cases from 2012 to 2016 (Figure 1A). It should be noted that morphine is a proxy for heroin abuse, as heroin itself is rarely detected in blood due to its rapid deacetylation into morphine *in vivo* (Karch, 2002).

The total number of drug deaths with a codeine-positive toxicology was approximately one-half that seen for morphine (Figure 1B and Supplementary Table S1). The primary source of codeine in these cases is, in all likelihood, the opium used in heroin production, as codeine is a known, albeit minor, constituent (∼1–2%) of opium (Karch, 2002). Consistent with this interpretation, essentially all (96%) of codeine-positive blood samples also contained morphine, whereas codeine was detected in only one-half of morphine-positive cases (Supplementary Table S1).

A large number of drug deaths involved hydrocodone, which, unlike morphine and codeine, was markedly consistent on a year-to-year basis from 2012 through 2016 (Figure 1C). Methadone-positive and oxycodone-positive cases, though fewer in number, were also relatively stable over time (Figures 1I,J). In contrast, drug deaths involving hydromorphone, though initially quite low, increased 10-fold over a period of several years, with a subsequent decline from the zenith in Q1 2015 (Figure 1D).

The illicit stimulant cocaine and the prescription benzodiazepine alprazolam are two of the drugs most commonly co-abused with opioids and associated with opioid deaths (Warner et al., 2016); this pattern of co-abuse was evident in our opioid-abusing cohort (Supplementary Table S1). Interestingly, although the incidence of alprazolam-positive cases increased by 60% from 2012 to 2016 (Figure 1K), the incidence of cocaine use in drug deaths increased 2.6-fold during the same period (Figure 1L).

Undoubtedly, the most striking change during this 5-year period pertained to detection in blood of the synthetic opioid fentanyl and its analogs. Fentanyl-positive cases (Figure 1E) were nearly absent for the first half of this period, but increased 33-fold by 2016. Acetylfentanyl (Figure 1F) first appeared in our cohort in 2015. Furanylfentanyl (Figure 1G) and the highly potent fentanyl analog, carfentanil (Figure 1H) first appeared in our cohort only in 2016, but the number of cases increased with subsequent quarters.

We next used decision tree (CHAID) analysis to predict the relationship between the presence of any individual drug and the likelihood of drug death. Every 2012–2016 WCMEO case with a quantifiable blood level of any drug or other chemical (i.e., not solely drugs of abuse; *N* = 7731) was scored for the presence or absence of each drug and the WCMEO determination of cause of death as a drug-related death or non-drug-related death (see section “Materials and Methods”). The stark temporal demarcation of fentanyl-associated deaths (Figure 1) led us to conduct CHAID analyses separately for 2012–2014 (prior to the bulk of fentanyl cases) and 2015–2016 (since the onset of the current fentanyl epidemic). For ease of visualization, the resulting decision trees were split into their two major branches (intact decision trees with additional details can be found in Supplementary Figures S1, S2).

**FIGURE 2:**
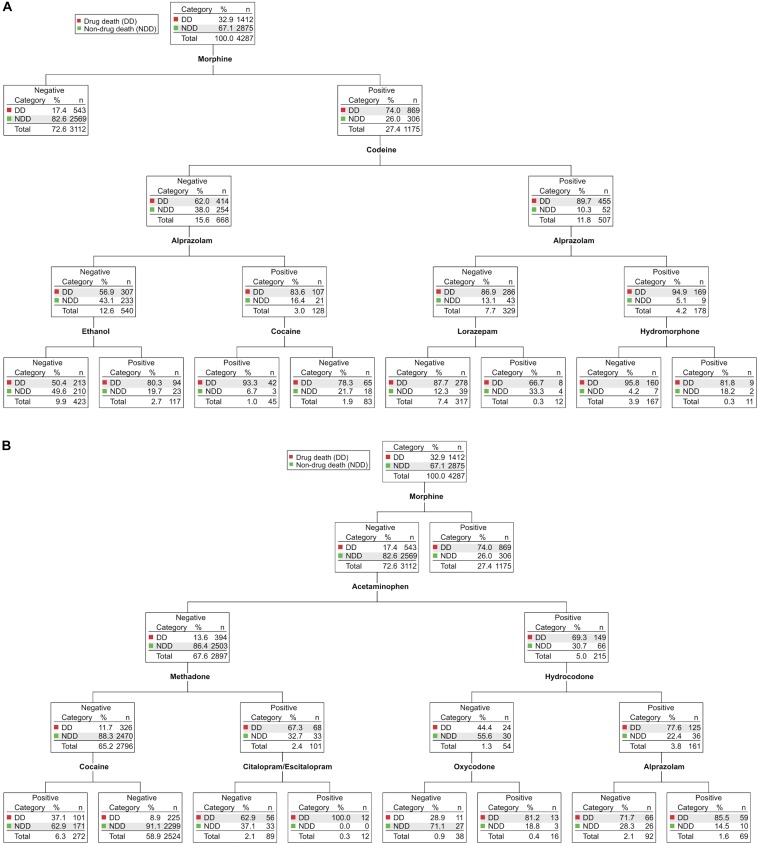
Chi-square Automatic Interaction Detector (CHAID) analyses of drug-related deaths and non-drug-related deaths in Wayne County from 2012–2014. Determination of the drugs and drug combinations most predictive of drug deaths (DD, red) vs. non-drug deaths (NDD, green). In each node, the total number of cases and their partitioning between DD and NDD (n, %) is shown. For ease of visualization, the major branches have been divided after the first parent node into morphine-positive cases **(A)** and morphine-negative cases **(B)**. Each node is statistically significant (minimum Bonferroni-adjusted *p* < 0.05). The full, intact decision tree is shown in Supplementary Figure S1.

Figure 2 shows CHAID data for 2012–2014 (*N* = 4287). The presence of morphine in blood (as stated above, a proxy for heroin use) was a highly significant (3.945E-270) predictor, such that 74% of morphine-positive cases were drug deaths whereas 83% of morphine-negative cases were non-drug deaths (Figure 2A, top). Further nodal branching of morphine-positive cases based on the presence or absence of other drugs led to no further segregation of drug deaths from non-drug deaths: morphine-positive cases were statistically much more likely to have died of drug abuse regardless of the presence or absence of other opioids (e.g., codeine, hydromorphone, hydrocodone, methadone, oxycodone) or non-opioid drugs of abuse (e.g., benzodiazepines, cocaine, ethanol; Figure 2A and Supplementary Figure S1).

Unexpectedly, in cases with morphine-negative toxicology (Figure 2B), the presence of the non-opioid analgesic acetaminophen was a highly significant (9.240E-96) biomarker for drug death: 69% of acetaminophen-positive cases were drug deaths, whereas 86% of acetaminophen-negative cases were non-drug deaths. The acetaminophen-positive branch of the decision tree has subsequent nodes for the presence/absence of hydrocodone and oxycodone (Figure 2B, right side), indicating that the predictive value of acetaminophen for drug deaths likely derives from the abuse of these common prescription opioid-acetaminophen combination formulations.

In the tree branch negative for both morphine and acetaminophen (Figure 2B, left side), the opioid methadone was a highly significant (7.473E-58) predictor of drug death. Though having relatively limited uses compared to other opioid drugs (e.g., in drug maintenance of opioid abuse disorders and treatment of severe chronic pain), methadone is associated with a disproportionate number of deaths, thought to be related to its long half-life, propensity to induce long QT syndrome and ventricular arrhythmias, and/or significant drug–drug interactions at the level of metabolism (Karch, 2002; Girardin et al., 2013). A subset of cases positive for both methadone and the commonly prescribed selective serotonin reuptake inhibitor (SSRI) antidepressant citalopram/escitalopram were, without exception, drug deaths (Figure 2B). Citalopram, which has a greater propensity to induce long QT syndrome than other SSRIs (Funk and Bostwick, 2013; Girardin et al., 2013), could increase methadone-related lethality at the level of induction of long QT syndrome and/or by increasing methadone levels via competitive inhibition of its hepatic metabolism (Karch, 2002; Funk and Bostwick, 2013; Girardin et al., 2013). Potential methadone interactions with citalopram warrant further investigation since these data suggest that their co-administration may be contraindicated.

Surprisingly, we observed that cases negative for morphine (a proxy for heroin), acetaminophen (a proxy for opioid/acetaminophen combination formulations), or methadone, but positive for cocaine (Figure 2B, bottom left), were significantly enriched (3.489E-43) in terms of non-drug-related deaths: two-thirds of the time these individuals did not die as a result of their cocaine use. Further, fully 94% of cases positive for both cocaine and levamisole were not drug deaths (1.943E-07; see Supplementary Figure S1, bottom left). Levamisole, a drug used primarily as a veterinary antihelmintic, is a common cocaine adulterant with a half-life shorter than of cocaine (Lee et al., 2012); thus, its detection is consistent with short, post-drug use survival time for these subjects. In fact, a review of individual case files confirmed that essentially all of these cocaine-positive, levamisole-positive cases were sudden non-drug deaths (40% homicides, 31% traumatic accidents, 21% suicides) proximal to final cocaine use (not shown).

**FIGURE 3:**
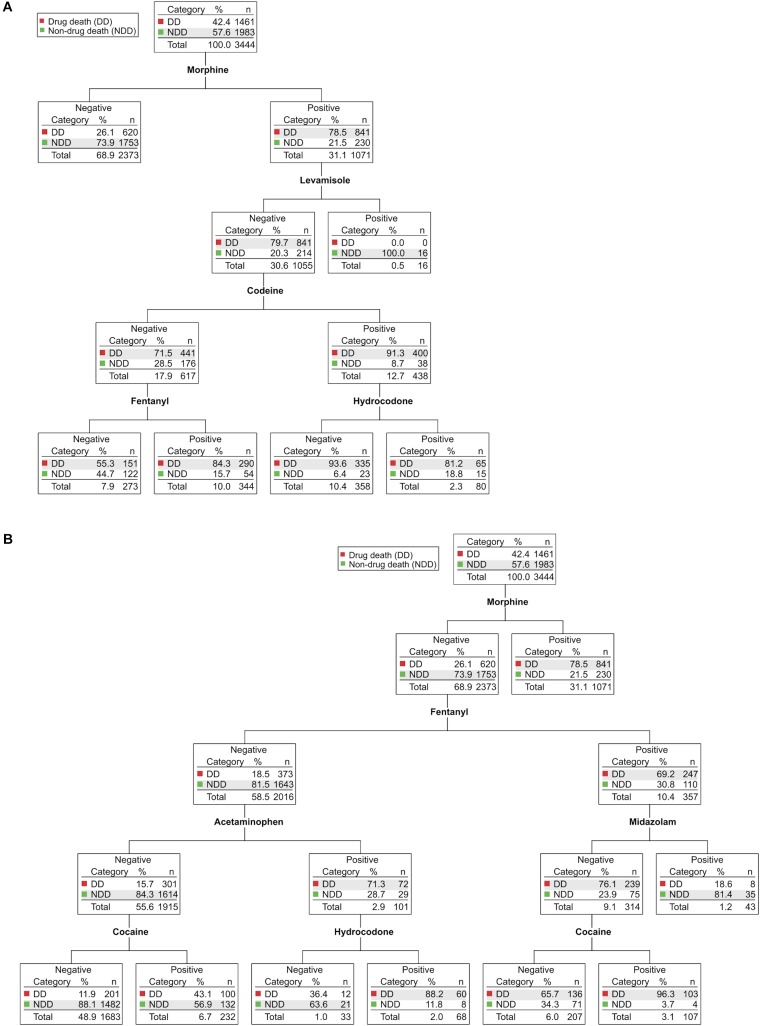
CHAID analyses of drug-related deaths and non-drug-related deaths in Wayne County from 2015–2016. Determination of the drugs and drug combinations most predictive of drug deaths (DD, red) vs. non-drug deaths (NDD, green). In each node, the total number of cases and their partitioning between DD and NDD (n, %) is shown. For ease of visualization, the major branches have been divided after the first parent node into morphine-positive cases **(A)** and morphine-negative cases **(B)**. Each node is statistically significant (minimum Bonferroni-adjusted *p* < 0.05). The full, intact decision tree is shown in Supplementary Figure S2.

Figure 3 shows the comparable CHAID analysis for the years 2015–2016 (*N* = 3444). Once again, the presence of morphine in the blood was the best predictor (2.118E-182) of a drug-related death (Figure 3A): 79% of morphine-positive cases were drug deaths, whereas 74% of morphine-negative cases were non-drug deaths. Morphine-positive cases were statistically more likely to be drug users regardless of which other opioid or non-opioid drugs of abuse were also present (Figure 3A and Supplementary Figure S2).

Undoubtedly, the most importance difference between the 2012–2014 and 2015–2016 CHAID analyses was the prominent appearance of fentanyl in the latter decision tree, as fentanyl now predicts drug death in both morphine-positive cases (2.353E-15; Figure 3A) and in morphine-negative cases (8.640E-90; Figure 3B). Approximately 60% of fentanyl-positive drug deaths were also positive for morphine (Supplementary Table S1), consistent with the common practice of adding non-pharmaceutical fentanyl to heroin as a means of reducing product cost and/or increasing potency (Algren et al., 2013). Nevertheless, we found that cases positive for fentanyl but negative for morphine were still primarily (69%) drug deaths, whereas cases negative for both morphine and fentanyl were largely (82%) not (Figure 3B).

Interestingly, a small subset of morphine-negative, fentanyl-positive cases was also positive for midazolam, a short-acting benzodiazepine used primarily for in-hospital procedures, often in combination with fentanyl. These cases were overwhelmingly (81%) not drug deaths (Figure 3B, right). Chart review revealed that they were, in fact, exclusively in-hospital deaths due to serious medical conditions (e.g., multi-organ failure, sepsis). The fraction of these cases classified as drug deaths involved drug self-ingestion as the precipitating event for subsequent hospitalization and fatal medical complications (not shown). The combination of fentanyl plus midazolam was, therefore, a highly significant (1.859E-14) blood biomarker for in-hospital death following serious medical illness and administration of these drugs for therapeutic purposes. This contrasts with the benzodiazepine alprazolam which, when found in combination with any of a number of opioids, is highly predictive of drug death (Figure 2).

In 2015–2016, in cases negative for both morphine and fentanyl (Figure 3B, left), the presence of acetaminophen was a highly statistical (1.229E-44) discriminator as to the likelihood of drug death (as had been seen in the 2012–2014 CHAID analysis). Branches downstream of acetaminophen-positive samples again revealed the presence of opioids that are most often sold in combination formulations (i.e., hydrocodone and hydromorphone; Figure 3B; see also Supplementary Figure S2). Likewise, as had been seen in the CHAID for prior years, the presence of the cocaine adulterant levamisole identified both morphine-positive cases (Figure 3A, 1.292E-14) and morphine-negative cases (Supplementary Figure S2, 4.403E-07) who were presumed drug users that did not die from drug use, once again consistent with the suggestion that levamisole provides a robust biomarker of cocaine use immediately prior to death.

Examining the more branched 2015–2016 CHAID tree presented in Supplementary Figure S2, one can also see the emergence of carfentanil as being highly diagnostic (1.228E-20) for drug death (bottom left), despite its quite recent appearance in Wayne County. Carfentanil is a fentanyl analog at least 100 times more potent than fentanyl, but not approved for medical use in humans (Misailidi et al., 2018). A preliminary analysis of interim data from 2017 indicates a continued trend for even greater numbers of fentanyl-associated drug deaths, the appearance of fentanyl analogs not previously detected in the WCMEO, and continued, simultaneous use of fentanyl and its analogs with heroin, cocaine, and other drugs of abuse (not shown).

## Conclusion

The data presented here resulted from systematic collection and unbiased statistical analyses of forensic toxicological results. They highlight the fact that continued abuse of prescription opioids, growing abuse of heroin, and a dangerously lethal infusion of fentanyl and its analogs account for most drug-based deaths in Wayne County, Michigan. This general trend mirrors that observed elsewhere in the United States, as does a renewed tendency for poly-drug abuse involving concurrent use of cocaine, benzodiazepines, or other drugs of abuse. The increasing prominence of fentanyl and its analogs is particularly alarming, as these opioids are harbingers of persistently increasing drug-related mortality. Left unabated, fentanyl and its more potent analogs could well displace heroin as the primary cause of drug-related deaths in Wayne County and nationwide.

The use of CHAID-based decision tree analyses provided new insights regarding specific drugs, drug combinations, and biomarkers in blood most predictive of cause of death or circumstances surrounding death. The presence of the non-opioid drug acetaminophen was highly predictive of drug-related deaths, likely reflecting the use of various combined acetaminophen-opioid formulations on the market. Detection of the short-lived cocaine adulterant levamisole was predictive of a short post-cocaine survival time preceding a sudden, often violent, non-drug death. The combination of the opioid methadone and the SSRI citalopram was universally associated with drug death, indicating the possibility of a drug–drug interaction with an effect on the heart and/or drug metabolism. Lastly, fentanyl, alongside the short-acting benzodiazepine midazolam, was diagnostic for death following serious illness and medical interventions that included administration of these drugs in a hospital setting. The extent to which the specific drugs, drug combinations, and blood biomarkers most predictive of drug death in Wayne County generalize to other areas of the United States remains to be determined, but is readily amenable to CHAID analysis.

Collectively, our results provide strong support for the use of forensic toxicology coupled with CHAID and similar analyses, not only in medico-legal death investigations, but also in a broader context as predictive surveillance tools that can inform public health and medical intervention strategies for substance abuse disorders and associated diseases (e.g., hepatitis A, B, and C, human immunodeficiency virus) that have far-reaching societal effects.

## Data Availability

All pertinent data for this work are included in the manuscript and Supplementary Material. Data not included but referred to in text or numerically summarized in graphs and tables can be requested by contacting the corresponding author.

## Author Contributions

MS, CLS, CJS, and MB designed the study. CJS collected the samples. MS, CLS, MR, and ST performed the statistical analyses. MS, ST, CJS, and MB interpreted the results, prepared the figures, and wrote the manuscript. All authors read, critically revised, and approved the manuscript.

## Conflict of Interest Statement

The authors declare that the research was conducted in the absence of any commercial or financial relationships that could be construed as a potential conflict of interest.

## Footnotes

1 www.nmslabs.com
