# Supplementary material for: Opioid Deaths: Trends, Biomarkers, and Potential Drug Interactions Revealed by Decision Tree Analyses
Source: Front Neurosci. 2018 Oct 23;12:728. doi: 10.3389/fnins.2018.00728 (PMC6206231; doi:10.3389/fnins.2018.00728)

SUPPLEMENTAL INFORMATION FOR

Opioid deaths: trends, biomarkers, and potential drug interactions revealed by decision tree analyses

Manal H. Saad[1], Candace L. Savonen[1], Matthew Rumschlag[1], Sokol V. Todi[1], Carl J. Schmidt[2,3], Michael J. Bannon[1]*

1- Wayne State University School of Medicine, Department of Pharmacology
2- University of Michigan School of Medicine, Department of Pathology
3- Wayne County Medical Examiner's Office

*Correspondence: 540 E. Canfield, Scott Hall, Rm. 3108, Detroit, MI 48201, USA.
Email: mbannon@med.wayne.edu

Supplemental Table 1. Determination of the extent of co-abuse in drug deaths involving opioids.
The number of cases positive for a specific opioid during 2012-2016 is shown at the top of each column. Below that number are the numbers of cases also positive for the drugs listed.

Supplemental Figure 1: CHAID analyses of drug deaths and non-drug deaths in Wayne County from 2012-2014. Determination of the drugs and drug combinations most predictive of drug deaths (DD, red) versus non-drug deaths (NDD, green). In each node, the total number of cases and their partitioning between DD and NDD (N, %) is shown. Each node is statistically significant (minimum Bonferoni-adjusted $p<0.05$).

Supplemental Figure 2: CHAID analyses of drug deaths and non-drug deaths in Wayne County from 2015-2016. Determination of the drugs and drug combinations most predictive of drug deaths (DD, red) versus non-drug deaths (NDD, green). In each node, the total number of cases and their partitioning between DD and NDD (N, %) is shown. Each node is statistically significant (minimum Bonferoni-adjusted $p<0.05$).

Supplemental Table 1

Morphine Pos. (N=1710)	
Codeine	855
Cocaine	594
Alprazolam	498
Ethanol	462
Fentanyl	457
Delta-9-THC	328
Hydrocodone	288
Diazepam	240
Clonazepam	172
Diphenhydramine	152
Dihydrocodeine/Hydrocodol	151
Hydromorphone	150
Citalopram/Escitalopram	124
Cyclobenzaprine	112
Acetaminophen	88
Quetiapine	86
Methadone	72
Sertraline	70
Oxycodone	57
No additional co-abused drugs	65

Codeine Pos. (N=890)	
Morphine	855
Alprazolam	302
Cocaine	297
Ethanol	258
Hydrocodone	162
Delta-9-THC	158
Fentanyl	143
Diazepam	129
Dihydrocodeine/Hydrocodol	93
Clonazepam	91
Diphenhydramine	84
Hydromorphone	76
Citalopram/Escitalopram	64
Cyclobenzaprine	56
Acetaminophen	55
Quetiapine	48
Sertraline	35
Methadone	29
Oxycodone	28
No additional co-abused drugs	0

Hydrocodone Pos. (N=596)	
Dihydrocodeine/Hydrocodol	379
Alprazolam	291
Morphine	288
Acetaminophen	251
Hydromorphone	189
Codeine	162
Diazepam	151
Ethanol	126
Cocaine	120
Delta-9-THC	110
Fentanyl	93
Clonazepam	88
Cyclobenzaprine	75
Diphenhydramine	60
Citalopram/Escitalopram	58
Quetiapine	52
Oxycodone	48
Sertraline	38
Methadone	30
No additional co-abused drugs	2

Hydromorphone Pos. (N=288)	
Hydrocodone	189
Dihydrocodeine/Hydrocodol	161
Morphine	150
Alprazolam	128
Acetaminophen	116
Codeine	76
Diazepam	59
Delta-9-THC	56
Fentanyl	55
Clonazepam	55
Ethanol	54
Cocaine	49
Diphenhydramine	34
Quetiapine	27
Citalopram/Escitalopram	24
Oxycodone	24
Cyclobenzaprine	23
Sertraline	22
Methadone	12
No additional co-abused drugs	1

Fentanyl Pos. (N=760)	
Morphine	457
Cocaine	314
Delta-9-THC	184
Ethanol	182
Alprazolam	164
Codeine	143
Diphenhydramine	95
Hydrocodone	93
Diazepam	87
Clonazepam	77
Hydromorphone	55
Dihydrocodeine/Hydrocodol	52
Cyclobenzaprine	49
Quetiapine	35
Citalopram/Escitalopram	29
Sertraline	29
Methadone	26
Oxycodone	22
Acetaminophen	21
No additional co-abused drugs	48

Methadone Pos. (N=188)	
Morphine	72
Alprazolam	72
Cocaine	34
Diazepam	33
Delta-9-THC	32
Hydrocodone	30
Codeine	29
Ethanol	28
Fentanyl	26
Clonazepam	24
Cyclobenzaprine	22
Diphenhydramine	21
Dihydrocodeine/Hydrocodol	18
Citalopram/Escitalopram	18
Quetiapine	14
Hydromorphone	12
Oxycodone	11
Acetaminophen	9
Sertraline	5
No additional co-abused drugs	15

Oxycodone Pos. (N=158)	
Alprazolam	80
Morphine	57
Acetaminophen	51
Hydrocodone	48
Diazepam	39
Delta-9-THC	38
Ethanol	32
Dihydrocodeine/Hydrocodol	29
Codeine	28
Clonazepam	28
Hydromorphone	24
Cocaine	23
Fentanyl	22
Diphenhydramine	21
Cyclobenzaprine	20
Citalopram/Escitalopram	17
Quetiapine	14
Sertraline	13
Methadone	11
No additional co-abused drugs	1

Counts of co-appearing drugs for top opioids

“N” is the total number of drug deaths that were positive for the respective opioid, including cases with “No additional co-abused drugs” from this list

Supplemental Figure 1

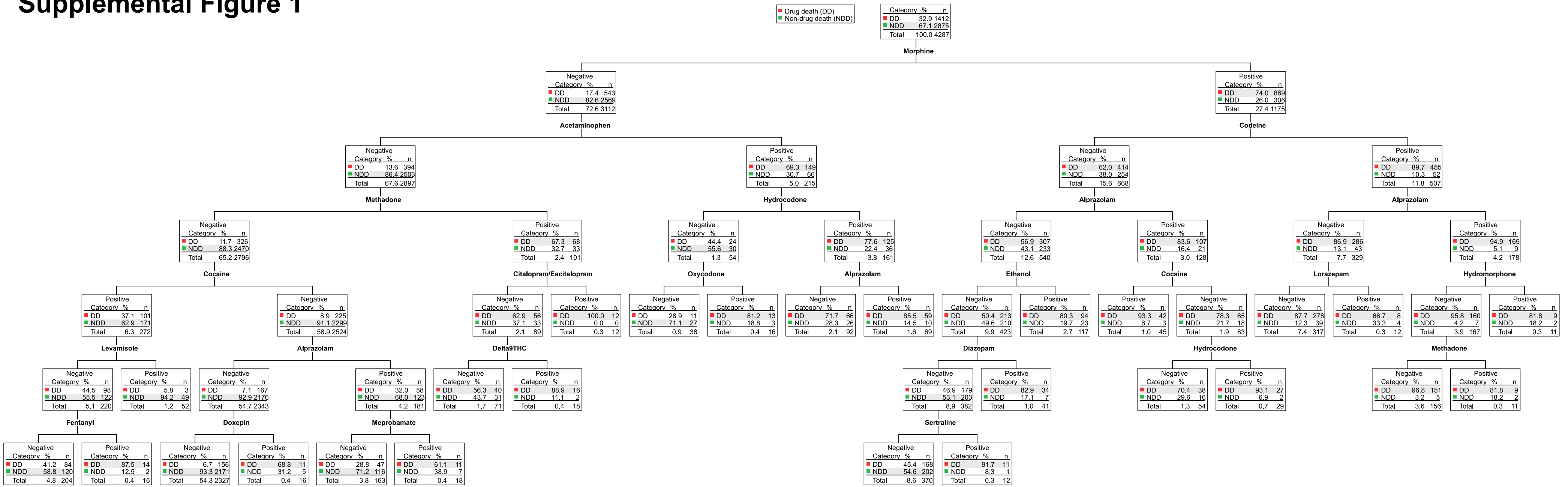

Supplemental Figure 2

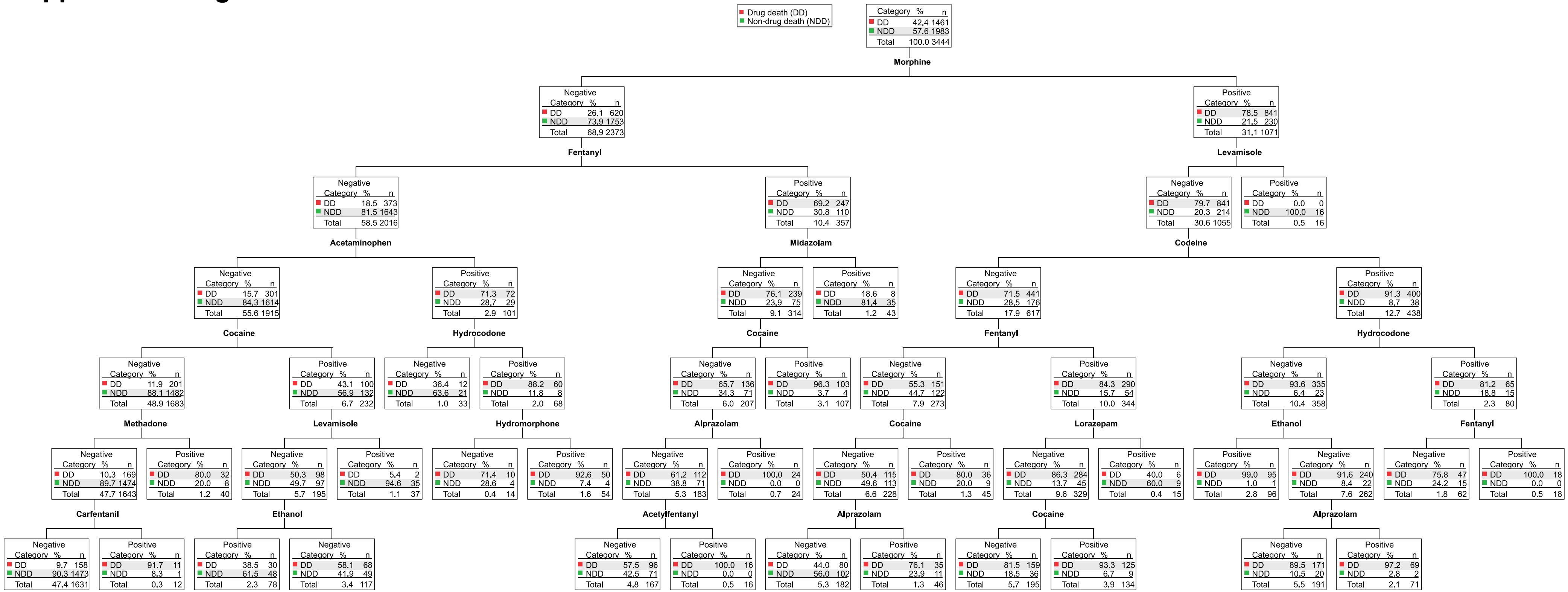

Supplement: Supplementary file 1 [file Data_Sheet_1.PDF]
